# Meta-analysis data for 104 Energy-Economy Nexus papers

**DOI:** 10.1016/j.dib.2017.04.023

**Published:** 2017-04-26

**Authors:** Vladimír Hajko, Agáta Kociánová, Martina Buličková

**Affiliations:** Department of Business Economics, Faculty of Business and Economics, Mendel University in Brno, Zemědělská 1, Brno, Czech Republic

**Keywords:** Energy, GDP, Energy-Economy Nexus

## Abstract

The data presented here are manually encoded characteristics of research papers in the area of Energy-Economy Nexus (empirical investigation of Granger causality between energy consumption and economic growth) that describe the methods, samples, and other details related to the individual estimations done in the examined empirical papers.

Data cover papers indexed by Scopus, published in economic journals, written in English, after year 2000. In addition, papers were manually filtered to only those that deal with Energy-Economy Nexus investigation and have at least 10 citations at (at the time of query – November 2015). This data are to be used to conduct meta-analysis – associated dataset was used in Hajko [1]. Early version of the dataset was used for multinomial logit estimation in Master thesis by Kociánová [2].

**Specifications Table**

**Table:** 

Subject area	*Economics*
More specific subject area	*Energy economics*
Type of data	*Table*
How data was acquired	*Manual encoding from research articles*
Data format	*Raw*
Experimental factors	
Experimental features	
Data source location	
Data accessibility	*Data is with this article.*

**Value of the data**

• Data cover 104 top-cited Energy-Economy Nexus articles.
• Suitable for meta-analysis.
• Recorded for studies as a whole (“aggregated dataset” has 1 observation= for 1 empirical study) and for individual estimation samples reported in the studies (“disaggregated dataset” has 1 observation= for 1 causality estimation in a paper).

## 1 Data

Table 1 provides the description and encoding values for the variables (note that variable descriptions are also available in the spreadsheet). The first line provides the full name, the second line a short name. Encoding values are represented by integer numbers (where appropriate, such as sample sizes, or number of individual estimations performed) or by categorical coding.

## 2 Experimental design, materials and methods

The data represent manually encoded characteristics of research papers in the area of Energy-Economy Nexus (empirical investigation of Granger causality between energy consumption and economic growth).

All covered papers are indexed by Scopus, published in economic journals, written in English, after year 2000. In addition, papers were manually filtered to only those that deal with Energy-Economy Nexus investigation and have at least 10 citations at (at the time of query – November 2015).

Variables describe both real-world characteristics and estimation characteristics used in the estimation samples in individual studies.

Original Scopus query:

( TITLE-ABS-KEY ( energy economy OR growth relationship OR causal OR nexus ) AND
PUBYEAR > 1999 ) AND
( causality OR cointegration ) AND
( LIMIT-TO ( SUBJAREA, "ECON" ) ) AND
( LIMIT-TO ( LANGUAGE, "English" ) ) AND
( LIMIT-TO ( SRCTYPE, "j" ) )

## Figures and Tables

**Fig. S1:**
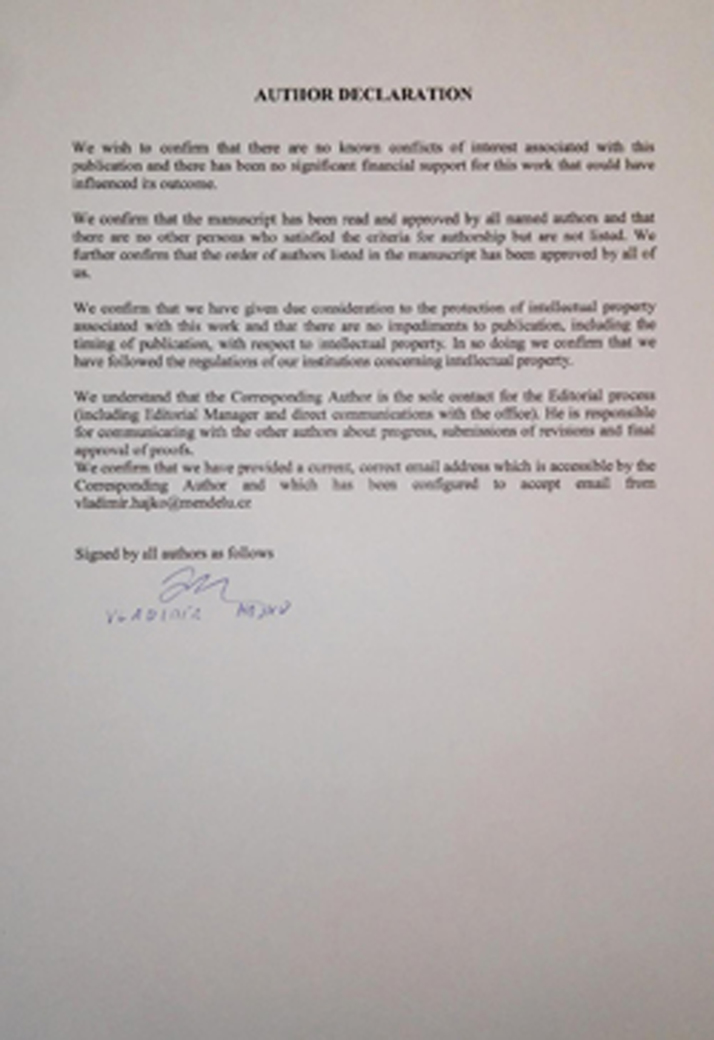


**Table 1:** Variable denominations and encoding values.

**Number of cross sections**	**Number of observations**	**Length of study in years**	**Sample size**
**NoCross**	**NoObs**	**LengthStudy**	**SampleSize**
Integer	Integer	Integer	**Integer**
**Year of publication**	**Number of citations**	**Number of separate individual estimates**	**USA only**
**PublicationYear**	**NoCitations**	**NoSepIndivEstim**	**USAonly**
Integer	Integer	Integer	0=No
			1=Yes
**China only**	**EU only**	**Panel data**	**Single region**
**Chinaonly**	**EUonly**	**PanelData**	**SingleRegion**
0=No	0=No	0=No	0=No
1=Yes	1=Yes	1=Yes	1=Yes
**Multiple energy types**	**Cointegration testing performed**	**Correction for multiple testing**	**Multivariate**
**MultiEnTypes**	**Cointegration**	**MultTestingCorr**	**Multivariate**
0=No	0=No	0=No	0=No
1=Yes	1=Yes	1=Yes	1=Yes
			2=Mixed
**Price as acontrol variable**	**EKC link**	**Structural breaks considered**	**Significance level used**
**PriceCtrl**	**EKCLink**	**StructBreaks**	**SignifLevel**
0=No	0=No	0=No	1=5/%
1=Yes	1=Yes	1=Yes	2=1/%
2=Mixed	2=Mixed	2=Mixed	3=Not specified/Ad hoc
**Sectoral**	**Variables per capita**	**Sign of causality considered**	**Frequency**
**Sectoral**	**VarsPerCap**	**CausalSign**	**Frequency**
1=Aggregate	1=total	1=No	1=Annual
2=Sectoral	2=per capita	2=In cointegration equation only/partially	2=Quarterly
3=Both	3=Both/Mixed	3=Yes	3=Monthly
		4=Inconclusive/unclear	4=Other
**Overall economic level**	**Basic causality direction concluded**	**Production control variables**	**Estimation method for causality**
**OverallEconLevel**	**CausalDirection**	**ProdCtrlVars**	**CausalMethod**
1=Low and lower-income economies	1=Neutrality (E×GDP)	1=None	1=Standard Granger causality (Engle-Granger, Sims)
2=Middle-income economies	2=Growth (E→GDP)	2=Capital	2=Modified Granger causality (Hsiao, Toda-Yamamoto)
3=High-income economies	3=Conservation (E←GDP)	3=Labor	3=ARDL
4=Mixed	4=Feedback (E↔GDP)	4=Both	4=Panel causality tests
	5=Mixed	5=Mixed	5=Other
**Geographic area**	**Cointegration method**	**Energy types**	
**Geoarea**	**CointegrationMethod**	**EnergyTypes**	
1=Mixed	1=None	1=Aggregate/all	
2=Asia (incl. Australia etc.)	2=Engle-Granger	2=Fossil fuels (oil, gas, coal)	
3=Europe	3=Johansen	3=Electricity	
4=Latin America /& the Caribbean	4=Panel - Pedroni/Larsson	4=Renewables	
5=Middle East and Africa	5=Other	5=Nuclear heat	
6=North America	6=Mixed	6=Exergy	
		7=Mixed	
