# Supplementary material for: Meta-analysis data for 104 Energy-Economy Nexus papers
Source: Data Brief. 2017 Apr 26;12:589–92. doi: 10.1016/j.dib.2017.04.023 (PMC5430162; doi:10.1016/j.dib.2017.04.023)
Supplement: Supplementary file 2 — Supplementary material [file mmc3.pdf]

Bibliography

Abalaba, B. and Dada, M. (2013). *Energy consumption and economic growth nexus: New empirical evidence from Nigeria*. International Journal of Energy Economics and Policy 3(4): 412–423.

Abid, M. and Sebri, M. (2012). *Energy consumption-economic growth nexus: Does the level of aggregation matter?* International Journal of Energy Economics and Policy 2(2): 55–62.

Acaravci, A. (2010). *Structural breaks,electricity consumption and economic growth:Evidence from turkey*. Romanian Journal of Economic Forecasting 13(2): 140–154.

Acaravci, A. and Ozturk, I. (2010). *Electricity consumption-growth nexus: Evidence from panel data for transition countries*. Energy Economics 32(3): 604–608. doi:10.1016/j.eneco.2009.10.016.

Adom, P. (2011). *Electricity Consumption-Economic growth nexus: The Ghanaian case*. International Journal of Energy Economics and Policy 1(1): 18–31.

Akinlo, A. (2008). *Energy consumption and economic growth: Evidence from 11 Sub-Sahara African countries*. Energy Economics 30(5): 2391–2400. doi:10.1016/j.eneco.2008.01.008.

Akinlo, A. (2009). *Electricity consumption and economic growth in Nigeria: Evidence from cointegration and co-feature analysis*. Journal of Policy Modeling 31(5): 681–693. doi:10.1016/j.jpolmod.2009.03.004.

Altinay, G. and Karagol, E. (2005). *Electricity consumption and economic growth: Evidence from Turkey*. Energy Economics 27(6): 849–856. doi:10.1016/j.eneco.2005.07.002.

Ang, J. B. (2008). *Economic development, pollutant emissions and energy consumption in Malaysia*. Journal of Policy Modeling 30(2): 271–278. doi:10.1016/j.jpolmod.2007.04.010.

Apergis, N. and Danuletiu, D. (2012). *Energy consumption and growth in Romania: Evidence from a panel error correction model*. International Journal of Energy Economics and Policy 2(4): 348–356.

Apergis, N. and Payne, J. E. (2009a). *Energy consumption and economic growth: Evidence from the Commonwealth of Independent States*. Energy Economics 31(5): 641–647. doi:10.1016/j.eneco.2009.01.011.

Apergis, N. and Payne, J. E. (2009b). *Energy consumption and economic growth in Central America: Evidence from a panel cointegration and error correction model*. Energy Economics 31(2): 211–216. doi:10.1016/j.eneco.2008.09.002.

Apergis, N. and Payne, J. E. (2010a). *Energy consumption and growth in South America: Evidence from a panel error correction model*. Energy Economics 32(6): 1421–1426. doi:10.1016/j.eneco.2010.04.006.

Apergis, N. and Payne, J. E. (2010b). *A panel study of nuclear energy consumption and economic growth*. Energy Economics 32(3): 545–549. doi:10.1016/j.eneco.2009.09.015.

Apergis, N. and Payne, J. E. (2010c). *Renewable energy consumption and growth in Eurasia*. Energy Economics 32(6): 1392–1397. doi:10.1016/j.eneco.2010.06.001.

Apergis, N. and Payne, J. E. (2011). *On the causal dynamics between renewable and non-renewable energy consumption and economic growth in developed and developing countries*. Energy Syst 2(3-4): 299–312. doi:10.1007/s12667-011-0037-6.

Apergis, N. and Payne, J. E. (2012). *Renewable and non-renewable energy consumption-growth nexus: Evidence from a panel error correction model*. Energy Economics 34(3): 733–738. doi:10.1016/j.eneco.2011.04.007.

Apergis, N., Payne, J. E., Menyah, K., and Wolde-Rufael, Y. (2010). *On the causal dynamics between emissions, nuclear energy, renewable energy, and economic growth*. Ecological Economics 69(11): 2255–2260. doi:10.1016/j.ecolecon.2010.06.014.

Asafu-Adjaye, J. (2000). *The relationship between energy consumption, energy prices and economic growth: time series evidence from Asian developing countries*. Energy Economics 22(6): 615–625. doi:10.1016/s0140-9883(00)00050-5.

Balcilar, M., Ozdemir, Z. A., and Arslanturk, Y. (2010). *Economic growth and energy consumption causal nexus viewed through a bootstrap rolling window*. Energy Economics 32(6): 1398–1410. doi:10.1016/j.eneco.2010.05.015.

Belke, A., Dobnik, F., and Dreger, C. (2011). *Energy consumption and economic growth: New insights into the cointegration relationship*. Energy Economics 33(5): 782–789. doi:10.1016/j.eneco.2011.02.005.

Bildirici, M. E. and Kayıkçı, F. (2012). *Economic growth and electricity consumption in former Soviet Republics*. Energy Economics 34(3): 747–753. doi:10.1016/j.eneco.2012.02.010.

Binh, P. (2011). *Energy consumption and economic growth in Vietnam: Threshold cointegration and causality analysis*. International Journal of Energy Economics and Policy 1(1): 1–17.

Bloch, H., Rafiq, S., and Salim, R. (2012). *Coal consumption, CO2 emission and economic growth in China: Empirical evidence and policy responses*. Energy Economics 34(2): 518–528. doi:10.1016/j.eneco.2011.07.014.

Bloch, H., Rafiq, S., and Salim, R. (2015). *Economic growth with coal, oil and renewable energy consumption in China: Prospects for fuel substitution.* Economic Modelling 44: 104–115. doi:10.1016/j.econmod.2014.09.017.

Bobinaite, V., Juozapaviciene, A., and Konstantinaviciute, I. (2011). *Assessment of Causality Relationship between Renewable Energy Consumption and Economic Growth in Lithuania.* Engineering Economics 22(5): 510–518. doi:10.5755/j01.ee.22.5.969.

Chiou-Wei, S. Z., Chen, C.-F., and Zhu, Z. (2008). *Economic growth and energy consumption revisited — Evidence from linear and nonlinear Granger causality.* Energy Economics 30(6): 3063–3076. doi:10.1016/j.eneco.2008.02.002.

Cleveland, C. J., Kaufmann, R. K., and Stern, D. I. (2000). *Aggregation and the role of energy in the economy.* Ecological Economics 32(2): 301–317. doi:10.1016/s0921-8009(99)00113-5.

Coers, R. and Sanders, M. (2013). *The energy–GDP nexus; addressing an old question with new methods.* Energy Economics 36: 708–715. doi:10.1016/j.eneco.2012.11.015.

Costantini, V. and Martini, C. (2010). *The causality between energy consumption and economic growth: A multi-sectoral analysis using non-stationary cointegrated panel data.* Energy Economics 32(3): 591–603. doi:10.1016/j.eneco.2009.09.013.

Damette, O. and Seghir, M. (2013). *Energy as a driver of growth in oil exporting countries?* Energy Economics 37: 193–199. doi:10.1016/j.eneco.2012.12.011.

Dergiades, T., Martinopoulos, G., and Tsoulfidis, L. (2013). *Energy consumption and economic growth: Parametric and non-parametric causality testing for the case of Greece.* Energy Economics 36: 686–697. doi:10.1016/j.eneco.2012.11.017.

Dogan, E. (2014). *Energy consumption and economic growth: Evidence from low-income countries in Sub-Saharan Africa.* International Journal of Energy Economics and Policy 4(2): 154–162.

Dritsaki, C. and Dritsaki, M. (2014). *Causal relationship between energy consumption, economic growth and CO2 emissions: A dynamic panel data approach.* International Journal of Energy Economics and Policy 4(2): 125–136.

Eddrief-Cherfi, S. and Kourbali, B. (2012). *Energy consumption and economic growth in Algeria: Cointegration and causality analysis.* International Journal of Energy Economics and Policy 2(4): 238–249.

Esso, L. J. (2010). *Threshold cointegration and causality relationship between energy use and growth in seven African countries.* Energy Economics 32(6): 1383–1391. doi:10.1016/j.eneco.2010.08.003.

Farhani, S. and Rejeb, J. (2012). *Energy consumption, economic growth and CO2 emissions: Evidence from panel data for MENA region.* International Journal of Energy Economics and Policy 2(2): 71–81.

Frame, B. and Brown, J. (2008). *Developing post-normal technologies for sustainability.* Ecological Economics 65(2): 225–241. doi:10.1016/j.ecolecon.2007.11.010.

Fuinhas, J. A. and Marques, A. C. (2012). *Energy consumption and economic growth nexus in Portugal, Italy, Greece, Spain and Turkey: An ARDL bounds test approach (1965–2009).* Energy Economics 34(2): 511–517. doi:10.1016/j.eneco.2011.10.003.

Gelo, T. (2009). *Causality between economic growth and energy consumption in Croatia.* Zbornik Radova Ekonomskog Fakultet au Rijeci 27(2): 327–348.

Georgantopoulos, A. (2012). *Electricity consumption and economic growth: Analysis and forecasts using VAR/VEC approach for Greece with capital formation.* International Journal of Energy Economics and Policy 2(4): 263–278.

Gross, C. (2012). *Explaining the (non-) causality between energy and economic growth in the U.S.—A multivariate sectoral analysis.* Energy Economics 34(2): 489–499. doi:10.1016/j.eneco.2011.12.002.

Hamit-Haggar, M. (2012). *Greenhouse gas emissions, energy consumption and economic growth: A panel cointegration analysis from Canadian industrial sector perspective.* Energy Economics 34(1): 358–364. doi:10.1016/j.eneco.2011.06.005.

Hondroyiannis, G., Lolos, S., and Papapetrou, E. (2002). *Energy consumption and economic growth: assessing the evidence from Greece.* Energy Economics 24(4): 319–336. doi:10.1016/s0140-9883(02)00006-3.

Ighodaro, C. A. U. (2010). *Co-integration and causality relationship between energy consumption and economic growth: Further empirical evidence for Nigeria.* Journal of Business Economics and Management 11(1): 97–111. doi:10.3846/jbem.2010.05.

Islam, F., Shahbaz, M., Ahmed, A. U., and Alam, M. M. (2013). *Financial development and energy consumption nexus in Malaysia: A multivariate time series analysis.* Economic Modelling 30: 435–441. doi:10.1016/j.econmod.2012.09.033.

Jafari, Y., Othman, J., and Nor, A. H. S. M. (2012). *Energy consumption, economic growth and environmental pollutants in Indonesia.* Journal of Policy Modeling 34(6): 879–889. doi:10.1016/j.jpolmod.2012.05.020.

Kahsai, M. S., Nondo, C., Schaeffer, P. V., and Gebremedhin, T. G. (2012). *Income level and the energy consumption–GDP nexus: Evidence from Sub-Saharan Africa.* Energy Economics 34(3): 739–746. doi:10.1016/j.eneco.2011.06.006.

Kalyoncu, H., Gürsoy, F., and Göcen, H. (2013). *Causality relationship between GDP and energy consumption in Georgia, Azerbaijan and Armenia.* International Journal of Energy Economics and Policy 3(1): 111–117.

Kaplan, M., Ozturk, I., and Kalyoncu, H. (2011). *Energy consumption and economic growth in Turkey: Cointegration and causality analysis.* Romanian Journal of Economic Forecasting 14(2): 31–41.

Kasman, A. and Duman, Y. S. (2015). *CO2 emissions, economic growth, energy consumption, trade and urbanization in new EU member and candidate countries: A panel data analysis.* Economic Modelling 44: 97–103. doi: 10.1016/j.econmod.2014.10.022.

Kayhan, S., Adiguzel, U., Bayat, T., and Lebe, F. (2010). *Causality relationship between real GDP and electricity consumption in Romania (2001-2010).* Romanian Journal of Economic Forecasting 13(4): 169–183.

Kwakwa, P. (2012). *Disaggregated energy consumption and economic growth in Ghana.* International Journal of Energy Economics and Policy 2(1): 34–40.

Lau, E., Chye, X.-H., and Choong, C.-K. (2011). *Energy-growth causality: Asian countries revisited.* International Journal of Energy Economics and Policy 1(4): 140–149.

Lee, C.-C. (2005). *Energy consumption and GDP in developing countries: A cointegrated panel analysis.* Energy Economics 27(3): 415–427. doi:10.1016/j.eneco.2005.03.003.

Lee, C.-C. and Chang, C.-P. (2008). *Energy consumption and economic growth in Asian economies: A more comprehensive analysis using panel data.* Resource and Energy Economics 30(1): 50–65. doi:10.1016/j.reseneeco.2007.03.003.

Lee, C.-C., Chang, C.-P., and Chen, P.-F. (2008). *Energy-income causality in OECD countries revisited: The key role of capital stock.* Energy Economics 30(5): 2359–2373. doi:10.1016/j.eneco.2008.01.005.

Lee, C.-C. and Chien, M.-S. (2010). *Dynamic modelling of energy consumption, capital stock, and real income in G-7 countries.* Energy Economics 32(3): 564–581. doi:10.1016/j.eneco.2009.08.022.

Lee, C.-C. and Chiu, Y.-B. (2011). *Nuclear energy consumption, oil prices, and economic growth: Evidence from highly industrialized countries.* Energy Economics 33(2): 236–248. doi:10.1016/j.eneco.2010.07.001.

Lise, W. and Montfort, K. V. (2007). *Energy consumption and GDP in Turkey: Is there a co-integration relationship?* Energy Economics 29(6): 1166–1178. doi:10.1016/j.eneco.2006.08.010.

Lorde, T., Waithe, K., and Francis, B. (2010). *The importance of electrical energy for economic growth in Barbados*. Energy Economics 32(6): 1411–1420. doi:10.1016/j.eneco.2010.05.011.

Menegaki, A. N. (2011). *Growth and renewable energy in Europe: A random effect model with evidence for neutrality hypothesis*. Energy Economics 33(2): 257–263. doi:10.1016/j.eneco.2010.10.004.

Menyah, K. and Wolde-Rufael, Y. (2010). *Energy consumption, pollutant emissions and economic growth in South Africa*. Energy Economics 32(6): 1374–1382. doi:10.1016/j.eneco.2010.08.002.

Mishra, V., Smyth, R., and Sharma, S. (2009). *The energy-GDP nexus: Evidence from a panel of Pacific Island countries*. Resource and Energy Economics 31(3): 210–220. doi:10.1016/j.reseneeco.2009.04.002.

Narayan, P. K. and Popp, S. (2012). *The energy consumption-real GDP nexus revisited: Empirical evidence from 93 countries*. Economic Modelling 29(2): 303–308. doi:10.1016/j.econmod.2011.10.016.

Narayan, P. K. and Singh, B. (2007). *The electricity consumption and GDP nexus for the Fiji Islands*. Energy Economics 29(6): 1141–1150. doi:10.1016/j.eneco.2006.05.018.

Naser, H. (2014). *Oil market, nuclear energy consumption and economic growth: Evidence from emerging economies*. International Journal of Energy Economics and Policy 4(2): 288–296.

Ocal, O., Ozturk, I., and Aslan, A. (2013). *Coal consumption and economic growth in Turkey*. International Journal of Energy Economics and Policy 3(2): 193–198.

Odhiambo, N. M. (2009). *Electricity consumption and economic growth in South Africa: A trivariate causality test*. Energy Economics 31(5): 635–640. doi:10.1016/j.eneco.2009.01.005.

Oh, W. and Lee, K. (2004a). *Causal relationship between energy consumption and GDP revisited: the case of Korea 1970–1999*. Energy Economics 26(1): 51–59. doi:10.1016/s0140-9883(03)00030-6.

Oh, W. and Lee, K. (2004b). *Energy consumption and economic growth in Korea: testing the causality relation*. Journal of Policy Modeling 26(8-9): 973–981. doi:10.1016/j.jpolmod.2004.06.003.

Omri, A. (2013). *CO2 emissions, energy consumption and economic growth nexus in MENA countries: Evidence from simultaneous equations models*. Energy Economics 40: 657–664. doi:10.1016/j.eneco.2013.09.003.

Onafowora, O. A. and Owoye, O. (2014). *Bounds testing approach to analysis of the environment Kuznets curve hypothesis*. Energy Economics 44: 47–62. doi:10.1016/j.eneco.2014.03.025.

Ouédraogo, I. M. (2010). *Electricity consumption and economic growth in Burkina Faso: A cointegration analysis.* Energy Economics 32(3): 524–531. doi: 10.1016/j.eneco.2009.08.011.

Ouedraogo, N. S. (2013). *Energy consumption and economic growth: Evidence from the economic community of West African States (ECOWAS).* Energy Economics 36: 637–647. doi:10.1016/j.eneco.2012.11.011.

Ozturk, I. and Uddin, G. (2012). *Causality among carbon emissions, energy consumption and growth in India.* Ekonomska Istrazivanja 25(3): 752–775.

Paul, B. P. and Uddin, G. S. (2011). *Energy and output dynamics in Bangladesh.* Energy Economics 33(3): 480–487. doi:10.1016/j.eneco.2010.11.011.

Paul, S. and Bhattacharya, R. N. (2004). *Causality between energy consumption and economic growth in India: a note on conflicting results.* Energy Economics 26(6): 977–983. doi:10.1016/j.eneco.2004.07.002.

Saatci, M. and Dumrul, Y. (2013). *The relationship between energy consumption and economic growth: Evidence from a structural break analysis for Turkey.* International Journal of Energy Economics and Policy 3(1): 20–29.

Sakiru Adebola, S. (2011). *Electricity consumption and economic growth: Trivariate investigation in Botswana with capital formation.* International Journal of Energy Economics and Policy 1(2): 32–46.

Sbia, R., Shahbaz, M., and Hamdi, H. (2014). *A contribution of foreign direct investment, clean energy, trade openness, carbon emissions and economic growth to energy demand in UAE.* Economic Modelling 36: 191–197. doi: 10.1016/j.econmod.2013.09.047.

Shahbaz, M., Khan, S., and Tahir, M. I. (2013). *The dynamic links between energy consumption, economic growth, financial development and trade in China: Fresh evidence from multivariate framework analysis.* Energy Economics 40: 8–21. doi:10.1016/j.eneco.2013.06.006.

Shahbaz, M., Zeshan, M., and Afza, T. (2012). *Is energy consumption effective to spur economic growth in Pakistan? New evidence from bounds test to level relationships and Granger causality tests.* Economic Modelling 29(6): 2310–2319. doi:10.1016/j.econmod.2012.06.027.

Shahiduzzaman, M. and Alam, K. (2012). *Cointegration and causal relationships between energy consumption and output: Assessing the evidence from Australia.* Energy Economics 34(6): 2182–2188. doi:10.1016/j.eneco.2012.03.006.

Soytas, U. and Sari, R. (2003). *Energy consumption and GDP: causality relationship in G-7 countries and emerging markets.* Energy Economics 25(1): 33–37. doi:10.1016/s0140-9883(02)00009-9.

Soytas, U. and Sari, R. (2006). *Can China contribute more to the fight against global warming?* Journal of Policy Modeling 28(8): 837–846. doi:10.1016/j.jpolmod.2006.06.016.

Soytas, U. and Sari, R. (2009). *Energy consumption, economic growth, and carbon emissions: Challenges faced by an EU candidate member*. Ecological Economics 68(6): 1667–1675. doi:10.1016/j.ecolecon.2007.06.014.

Soytas, U., Sari, R., and Ewing, B. T. (2007). *Energy consumption, income, and carbon emissions in the United States*. Ecological Economics 62(3-4): 482–489. doi:10.1016/j.ecolecon.2006.07.009.

Squalli, J. (2007). *Electricity consumption and economic growth: Bounds and causality analyses of OPEC members*. Energy Economics 29(6): 1192–1205. doi:10.1016/j.eneco.2006.10.001.

Stern, D. I. and Enflo, K. (2013). *Causality between energy and output in the long-run*. Energy Economics 39: 135–146. doi:10.1016/j.eneco.2013.05.007.

Tiwari, A. (2011). *Primary Energy Consumption, CO2 Emissions and Economic Growth: Evidence from India*. South East European Journal of Economics and Business 6(2): 99–117. doi:10.2478/v10033-011-0019-6.

Tsani, S. Z. (2010). *Energy consumption and economic growth: A causality analysis for Greece*. Energy Economics 32(3): 582–590. doi:10.1016/j.eneco.2009.09.007.

Tugcu, C. T., Ozturk, I., and Aslan, A. (2012). *Renewable and non-renewable energy consumption and economic growth relationship revisited: Evidence from G7 countries*. Energy Economics 34(6): 1942–1950. doi:10.1016/j.eneco.2012.08.021.

Ucan, O., Aricioglu, E., and Yucel, F. (2014). *Energy consumption and economic growth nexus: Evidence from developed countries in Europe*. International Journal of Energy Economics and Policy 4(3): 411–419.

Vlahinić-Dizdarevi'c, N. and Ẑiković, S. (2010). *The role of energy in economic growth: The case of Croatia*. Zbornik Radova Ekonomskog Fakultet au Rijeci 28(1): 35–60.

Wolde-Rufael, Y. (2005). *Energy demand and economic growth: The African experience*. Journal of Policy Modeling 27(8): 891–903. doi:10.1016/j.jpolmod.2005.06.003.

Wolde-Rufael, Y. (2009). *Energy consumption and economic growth: The experience of African countries revisited*. Energy Economics 31(2): 217–224. doi:10.1016/j.eneco.2008.11.005.

Wolde-Rufael, Y. (2014). *Electricity consumption and economic growth in transition countries: A revisit using bootstrap panel Granger causality analysis*. Energy Economics 44: 325–330. doi:10.1016/j.eneco.2014.04.019.

Wolde-Rufael, Y. and Menyah, K. (2010). *Nuclear energy consumption and economic growth in nine developed countries*. Energy Economics 32(3): 550–556. doi:10.1016/j.eneco.2010.01.004.

Yang, Z. and Zhao, Y. (2014). *Energy consumption, carbon emissions, and economic growth in India: Evidence from directed acyclic graphs*. Economic Modelling 38: 533–540. doi:10.1016/j.econmod.2014.01.030.

Yıldırım, E., Sukruoglu, D., and Aslan, A. (2014). *Energy consumption and economic growth in the next 11 countries: The bootstrapped autoregressive metric causality approach*. Energy Economics 44: 14–21. doi:10.1016/j.eneco.2014.03.010.

Yuan, J., Zhao, C., Yu, S., and Hu, Z. (2007). *Electricity consumption and economic growth in China: Cointegration and co-feature analysis*. Energy Economics 29(6): 1179–1191. doi:10.1016/j.eneco.2006.09.005.

Zamani, M. (2007). *Energy consumption and economic activities in Iran*. Energy Economics 29(6): 1135–1140. doi:10.1016/j.eneco.2006.04.008.

Zhang, C. and Xu, J. (2012). *Retesting the causality between energy consumption and GDP in China: Evidence from sectoral and regional analyses using dynamic panel data*. Energy Economics 34(6): 1782–1789. doi:10.1016/j.eneco.2012.07.012.
